# Factors underlying COVID-19 vaccine and booster hesitancy and refusal, and incentivizing vaccine adoption

**DOI:** 10.1371/journal.pone.0274529

**Published:** 2022-09-22

**Authors:** Neil G. Bennett, David E. Bloom, Maddalena Ferranna

**Affiliations:** 1 CUNY Institute for Demographic Research, City University of New York, New York, New York, United States of America; 2 Austin W. Marxe School of Public and International Affairs, Baruch College, City University of New York, New York, New York, United States of America; 3 PhD Program in Sociology, CUNY Graduate Center, City University of New York, New York, New York, United States of America; 4 Department of Epidemiology and Biostatistics, CUNY Graduate School of Public Health & Health Policy, City University of New York, New York, New York, United States of America; 5 Department of Global Health and Population, Harvard T.H. Chan School of Public Health, Boston, Massachusetts, United States of America; 6 Department of Pharmaceutical and Health Economics, USC School of Pharmacy, Los Angeles, California, United States of America; Iowa State University, UNITED STATES

## Abstract

The paper investigates the factors underlying COVID-19 vaccine and booster hesitancy in the United States, and the efficacy of various incentives or disincentives to expand uptake. We use cross-sectional, national survey data on 3,497 U.S. adults collected online from September 10, 2021 to October 20, 2021 through the Qualtrics platform. Results from a multinomial logistic regression reveal that hesitancy and refusal were greatest among those who expressed a lack of trust either in government or in the vaccine’s efficacy (hesitancy relative risk ratio, or RRR: 2.86, 95% CI: 2.13–3.83, p<0.001). Hesitancy and refusal were lowest among those who typically get a flu vaccine (hesitancy RRR: 0.28, 95% CI: 0.21–0.36, p<0.001; refusal RRR: 0.08, 95% CI: 0.05–0.13, p<0.001). Similar results hold for the intention to get a booster shot among the fully vaccinated. Monetary rewards (i.e., lottery ticket and gift cards) fared poorly in moving people toward vaccination. In contrast, the prospect of job loss or increased health insurance premiums was found to significantly increase vaccine uptake, by 8.7 percentage points (p<0.001) and 9.4 percentage points (p<0.001), respectively. We also show that the motivations underlying individuals’ hesitancy or refusal to get vaccinated vary, which, in turn, suggests that messaging must be refined and directed accordingly. Also, moving forward, it may be fruitful to more deeply study the intriguing possibility that expanding flu vaccine uptake may also enhance willingness to vaccinate in times of pandemics. Last, disincentives such as work-based vaccination mandates that would result in job loss or higher health insurance premiums for those who refuse vaccination should be strongly considered to improve vaccine uptake in the effort to address the common good.

## Introduction

Despite the widespread availability of effective COVID-19 vaccines, many individuals still hesitate to take the vaccine or refuse outright to do so. For example, as of the end of August 2022, the proportion of U.S. individuals who were fully vaccinated was 67.5% and who received a booster shot was 32.8% (or 48.5% of the fully vaccinated) [1]. Unvaccinated individuals give various reasons for their hesitancy or opposition, including health, moral, religious, or even scientific ones. Certainly, this reluctance or refusal leaves many of them nakedly exposed to infection and to spreading that infection to others.

As vaccination uptake has hit stumbling blocks during the course of the pandemic, governments and businesses have sought either to incentivize adoption or to impose disincentives for refusing vaccination. Examples include the $1 million lottery created by the State of Ohio in May 2021 for randomly selected vaccine recipients, and Québec’s vaccine passport requirement, begun in January 2022, to enter Société des alcools du Québec and Société québécoise du cannabis locations. Analyses of the Ohio lottery found that its impact on vaccine adoption was minimal at best [2, 3]. In contrast, after the announcement by Québec Health Minister, Christian Dubé, daily appointments for first doses quadrupled in anticipation of the deadline [4].

Whether monetary or nonmonetary incentives can effectively increase COVID-19 vaccination uptake is controversial. Although financial incentives have been shown to encourage healthy behavior [5], sometimes they are ineffective or even backfire [6, 7]. Applied to COVID-19 vaccination, monetary incentives may be perceived as a bribe, or as a signal that the vaccine is ineffective or unsafe. Indeed, studies find little evidence that financial rewards increase COVID-19 vaccination uptake among the vaccine hesitant [8–10].

Mandatory COVID-19 certification to access public venues, workplaces, and nonessential businesses seems to be more effective at increasing vaccination [11] through the threat of a monetary loss, or the decline of physical or emotional well-being, but only if carefully designed [12]. Generally speaking, much research shows that motivation stemming from losses is greater than that generated from comparable gains [13].

We report findings from an online survey administered in September–October 2021 to shed light on the factors underlying vaccine and booster hesitancy and refusal in the United States and to investigate the potential effectiveness of various incentives and disincentives for improving vaccine adoption. We consider monetary and nonmonetary incentives, ranging from gift cards and the chance of winning a lottery, to increased healthcare costs, employers’ vaccine mandates, and the emergence of more transmissible and dangerous variants. Results from our study can help governments, businesses, and other organizations in designing effective strategies to increase COVID-19 vaccine uptake.

## Methods

### Data

Data for this study are drawn from a cross-sectional survey of 3,497 adults who reside in the United States conducted from September 10, 2021, to October 20, 2021. The survey collected data on individuals’ experience with COVID-19 and on individuals’ opinions and decisions about COVID-19 vaccination (To obtain these data, please go to https://www.hsph.harvard.edu/pgda/data/). The survey was administered online by Qualtrics, a survey platform with access to a convenience panel of several million U.S. adults who choose to participate in surveys in exchange for modest remuneration.

Qualtrics recruits panel members from various sources, including website intercept recruitment, member referrals, targeted email lists, gaming sites, customer loyalty web portals, permission-based networks, and social media. The panel members’ names, addresses, and dates of birth are typically validated via third-party verification prior to their joining the panel. Qualtrics compensates respondents in various ways, such as gift cards or airline miles, and prior to taking the survey participants are informed how they will be compensated.

We provided Qualtrics with quotas for gender, age, race/ethnic group, educational attainment, and region based on the 2015–2019 American Community Survey (ACS). We oversampled individuals belonging to racial and ethnic minorities (i.e., Hispanics and non-White racial groups) to enhance the robustness of the analysis. Table 1 in S1 File summarizes key demographic variables in comparison with data from the 2015–2019 ACS. The overall sample was closely representative of the U.S. population by age, gender, educational attainment, and region. The sample was skewed towards less-educated women and white men (Table 2 in S1 File), and thus we constructed post-stratification weights to align the sample with the U.S. gender-education-specific population distribution, as given by the 2015–2019 ACS.

Out of the 15,878 individuals invited to participate in the survey, 4,166 (26.2%) refused to participate, 7690 (48.4%) were rejected for overfilling quotas, violation of the speeder limit (i.e., survey completed in less than 5 minutes), or eligibility criteria, and 525 (3.3%) dropped out before completing the survey. The average time for survey completion was 14.25 minutes. All participants provided informed consent electronically before completing the survey.

### Outcomes

We partitioned respondents into three categories: partially or fully vaccinated (with the initial vaccination protocol), unwilling to get vaccinated, and hesitant or undecided. We inferred individuals were unwilling to get vaccinated if they were not partially or fully vaccinated and answer “Definitely no” to the question, “Do you intend to get vaccinated?” We defined “undecided” as individuals who were not vaccinated and answered “Definitely yes,” “Probably yes,” “I’m not sure,” or “Probably no” to the previous question. In addition, we inferred vaccinated individuals were hesitant or refused to take the booster shot if they answered “Probably yes,” “I’m not sure,” “Probably no,” or “Definitely no” to a question about their willingness to take the booster shot.

We considered a relatively wide range of monetary and nonmonetary incentives to increase vaccination uptake among those who are not vaccinated:

- A $100 or $200 gift card;
- $100 or $200 worth of tickets for a $1 million lottery;
- Proof of vaccination required from the employer as a condition of continued employment;
- Employer requirement that all unvaccinated employees be tested once a week for COVID-19 infection, with a cost to them of $10 per week;
- $200 health insurance cost increase per month if unvaccinated;
- Wide circulation in the United States of a substantially more transmissible and dangerous variant of COVID-19 (the details of which were not given).

We assess the impact of each incentive on the likelihood of getting vaccinated by comparing individuals’ intentions to get vaccinated with the incentive and their baseline intentions to get vaccinated (with intentions ranging from “1. Definitely not” to “5. Definitely yes” and a lower score representing more hesitancy).

### Statistical analyses

To assess the factors associated with vaccine hesitancy/refusal, we fit multinomial logistic regression models with either initial vaccination protocol hesitancy/refusal or booster shot hesitancy/refusal as the outcome and the following set of predictors: i) demographic/socioeconomic variables (age, gender, education, race/ethnicity, region, urbanicity, income, health insurance coverage, labor force participation, and household composition), ii) health status, iii) attitudes and beliefs (political affiliation, trust, religiosity, and perception of COVID-19 risk), and iv) experience with COVID-19. The overall effectiveness of incentives at increasing the willingness to get vaccinated was assessed by performing t-tests. Analyses were conducted using Stata, version 15.1.

## Results

### Descriptive statistics

Overall, 67.6% of the sample were partially or fully vaccinated (95% CI: 65.8–69.4), 18.3% were undecided (95% CI: 16.8–19.8), and 14.1% were unwilling (95% CI: 12.7–15.5) (Table 3 in S1 File). This is in line with data from the Kaiser Family Foundation COVID-19 Vaccine Monitor [14] from the same period (72% of U.S. adults with at least one dose of COVID-19 vaccine in October 2021). Among the most vaccinated subgroups were individuals aged 65 and older (89.2%, 95% CI: 86.6–91.7), people with a college degree (87.4%, 95% CI: 85.1–89.7), those who voted for Biden in the 2020 Presidential election (85.1%, 95% CI: 83.1–87.1), and individuals who typically get the flu vaccine (86.6%, 95% CI: 84.8–88.4). Among the least vaccinated subgroups were those without health insurance (42.9%, 95% CI: 37.7–48.1), those who knew someone who experienced severe side effects from the vaccine (56.6%, 95% CI: 51.9–61.3), and individuals with before-tax household income less than $50,000 per year (60.6%, 95% CI: 58.0–63.2). In addition, 60.2% of the fully vaccinated report an intention to get the booster shot (95% CI: 57.8–62.6) (Table 4 in S1 File).

### Vaccine hesitancy or refusal among the unvaccinated

**Fig 1:**
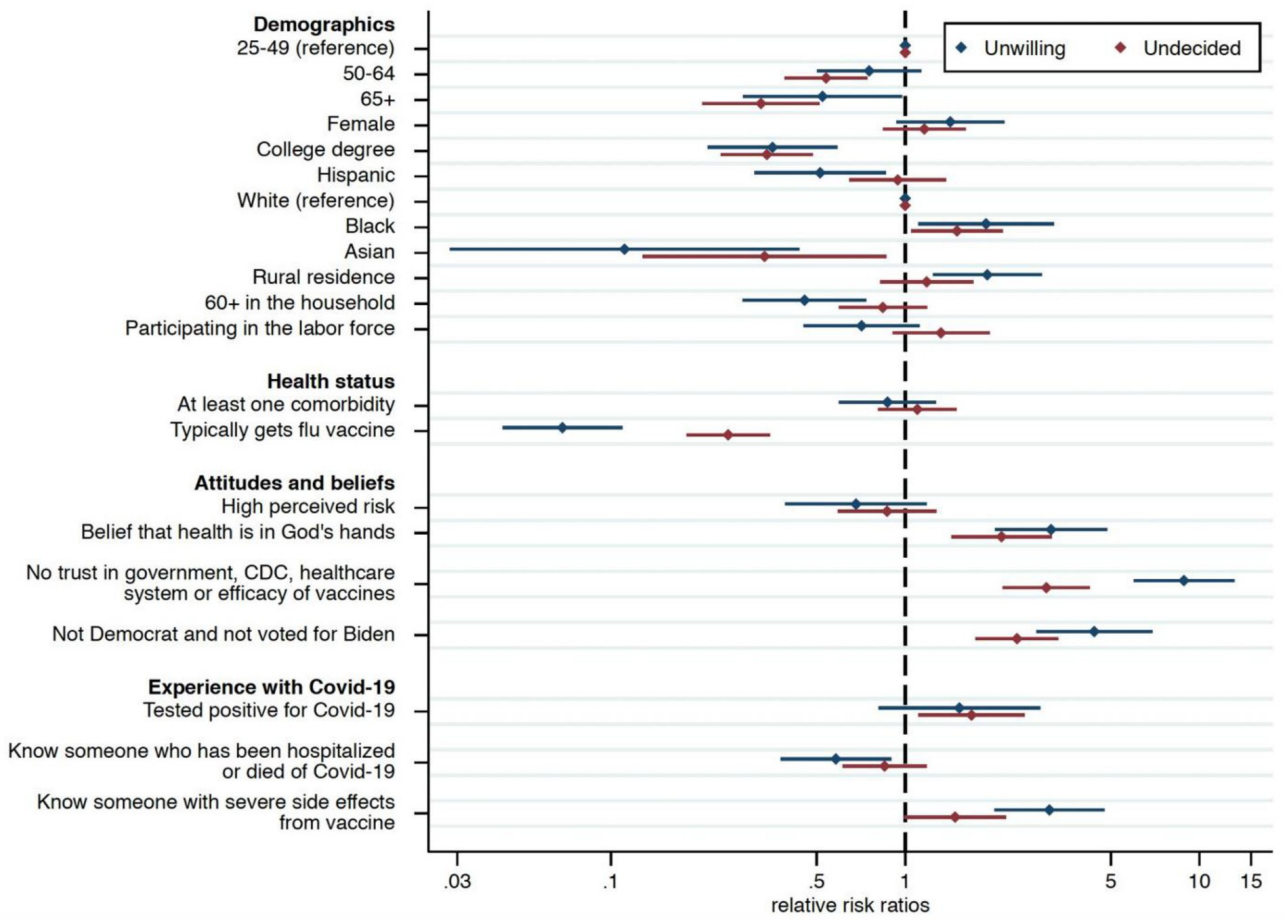
Factors associated with vaccine hesitancy and refusal among individuals aged 25 and older. *Notes*: Number of partially or fully vaccinated individuals = 2,232; number of individuals unwilling to get vaccinated = 350; number of individuals undecided = 519. Relative risk ratios (RRR) from multinomial logistic regression model predicting vaccine hesitancy (“undecided”) and refusal (“unwilling”) relative to those partially or fully vaccinated. The x-axis adopts a logarithmic scale. Weighted model. Inclusion of a dummy variable for respondents with missing income information (4.48% of the 25+ sample has missing income information) and of regional, labor force participation, and income covariates. Table 5 in S1 File shows regression results. The lines identify the 95% confidence intervals.

Fig 1 reports results from a multinomial logistic regression model estimating the relative risk ratios (RRR) of vaccine hesitancy and refusal relative to those who are either fully or partially vaccinated for individuals ages 25 and older. (Results are robust to the inclusion of the younger age group, 18–24, and to the use of weights, see Table 5 in S1 File).

Both vaccine hesitancy and rejection decrease monotonically with age, and are substantially less common among those with a college degree (hesitancy RRR: 0.32, 95% CI: 0.23–0.45, p<0.001; refusal RRR: 0.31, 95% CI: 0.19–0.51, p<0.001), Asians (hesitancy RRR: 0.35, 95% CI: 0.16–0.73, p = 0.006; refusal RRR: 0.17, 95% CI: 0.04–0.66, p = 0.011), and those aged 65 and older (hesitancy RRR: 0.30, 95% CI: 0.19–0.47, p<0.001; refusal RRR: 0.50, 95% CI: 0.27–0.93, p = 0.029). Respondents who were Hispanic (RRR: 0.49, 95% CI: 0.30–0.78, p = 0.003), whose household included someone at least 60 years of age (RRR: 0.53, 95% CI: 0.34–0.82, p = 0.004), and who knew someone who had been hospitalized or died of COVID-19 (RRR: 0.58, 95% CI: 0.40–0.84, p = 0.004) were less likely to be unwilling to be vaccinated. Refusal was more likely among those who knew someone whose vaccine side effects, in their estimation, were severe (RRR: 2.68, 95% CI: 1.79–4.01, p<0.001).

Rural residents were more likely to be unwilling to vaccinate (RRR: 1.60, 95% CI: 1.09–2.36, p = 0.017), a result consistent with recent research by the Centers for Disease Control and Prevention (CDC), perhaps due to poorer access to healthcare services, among other factors. The popular press has speculated that the urban-rural divide is rooted in differences in political ideology, but the results reported here are derived in a multivariable framework that controls separately for political ideology [15].

We also find that Blacks (hesitancy RRR: 1.64, 95% CI: 1.18–2.27, p = 0.003; refusal RRR: 1.89, 95% CI: 1.20–2.99, p = 0.006) and non-Democrats who did not vote for President Biden (hesitancy RRR: 2.27, 95% CI: 1.71–3.00, p<0.001; refusal RRR: 4.42, 95% CI: 2.87–6.77, p<0.001) were more likely to refuse the vaccine or to be hesitant and that differences in income did not explain vaccination status.

Vaccination hesitancy and refusal were much higher among respondents who believed that one’s health is in God’s hands (hesitancy RRR: 2.05, 95% CI: 1.45–2.91, p<0.001; refusal RRR: 2.54, 95% CI: 1.67–3.88, p<0.001) and among those who lack trust in the government, CDC, healthcare system, or efficacy of the vaccines (hesitancy RRR: 2.86, 95% CI: 2.13–3.83, p<0.001; refusal RRR: 9.07, 95% CI: 6.40–12.86, p<0.001). Although it may be the case that there is overlap among respondents in these two categories, it is interesting to note the very substantial association each has individually with attitudes towards vaccination.

Importantly, people who typically received a flu vaccine were substantially more likely to have taken the COVID-19 vaccine (hesitancy RRR: 0.28, 95% CI: 0.21–0.36, p<0.001; refusal RRR: 0.08, 95% CI: 0.05–0.13, p<0.001). Put simply, the sample’s descriptive statistics tell us that 10.5% of those age 25 and older who typically take the flu vaccine each year did not take the COVID-19 vaccine. In stark contrast, among the corresponding group who do not typically get vaccinated for the flu nearly half, 48.2%, did not avail themselves of the COVID-19 vaccine. Thus, although we cannot assume a causal relationship, fostering general vaccination habits may have positive spillover effects.

**Fig 2:**
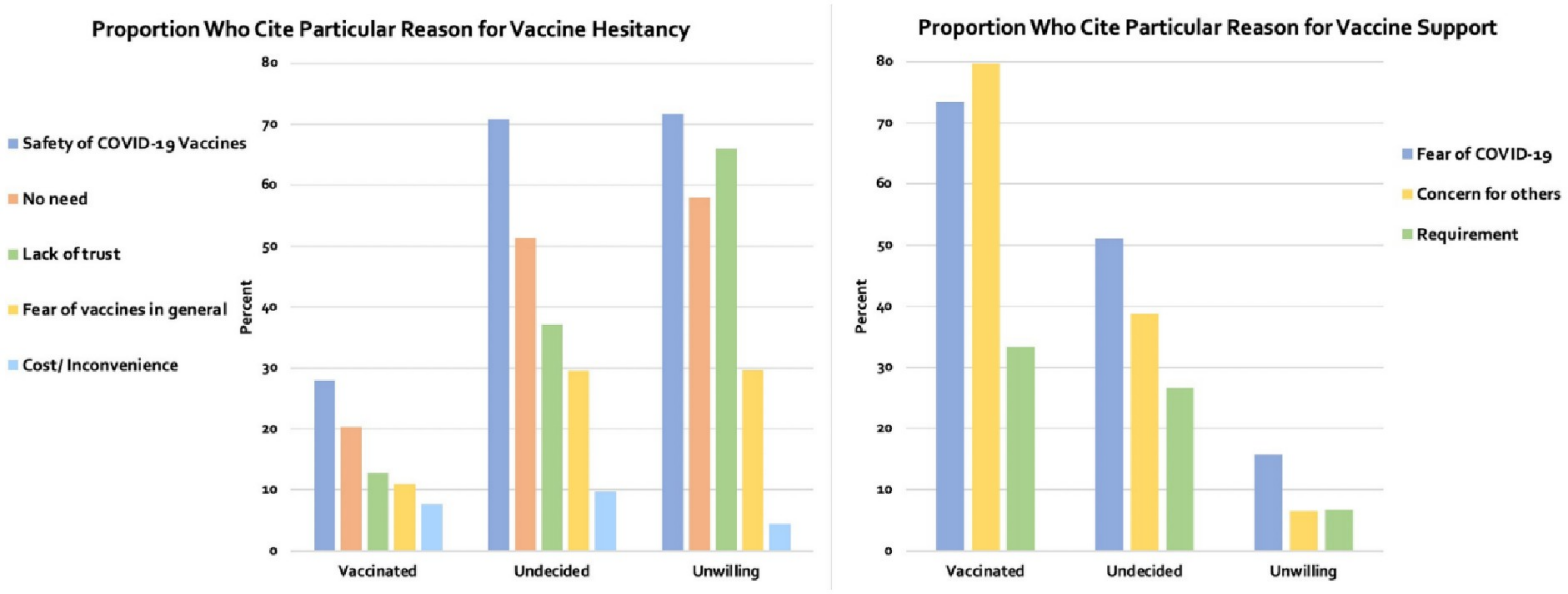
Vaccine hesitancy/acceptance by type of reason and vaccination status (%). *Notes*: For each vaccination category (partially or fully vaccinated, hesitant or undecided, and unwilling), the height of the bars represents the (weighted) percentage of respondents who select a particular reason. Table 6 in S1 File displays the results.

To better understand the reasons underlying vaccine acceptance and vaccine hesitancy or refusal, we asked respondents why they thought getting vaccinated would be unnecessary or a bad idea and why they thought being vaccinated would be good. We classified the negative reasons into five groups (“No need,” “Lack of trust,” “Cost/Inconvenience,” “Safety of COVID-19 vaccines,” and “Fear of vaccines in general.”), and the positive reasons into three groups (“Fear of COVID-19,” “Concern for others,” and “Requirement”). Table 1 summarizes the construction of these indexes, while Fig 2 displays the proportion of vaccinated and unvaccinated people who cite at least one reason for vaccine hesitancy or support within each category.

**Table 1:** Classification of factors underlying vaccine acceptance and vaccine hesitancy or refusal.

Reasons for accepting the vaccine	Reasons for being hesitant or refusing the vaccine
**Fear of COVID-19** I’m concerned about traveling using subways, buses, airplanes, taxis, etc. I’m afraid of hospitalization due to the virus. I’m afraid of long-term illness (e.g., including “long COVID”) due to the virus. I’m afraid of death due to the virus. **Concern for others** I want my family to be protected from the virus. I believe it’s our public duty to get vaccinated. **Requirement** My workplace requires or may require the vaccine. I think I may be required to get the vaccine to get into restaurants or bars, sporting/entertainment events, etc.	**No need** I’m not at high risk for COVID-19. I don’t think COVID-19 is a serious illness. I prefer to use masks and other precautions instead. I had COVID-19, and I believe I’m immune. I believe my health and safety are in God’s hands. **Lack of trust** I don’t think the vaccine will work. I don’t trust the government, the CDC, or the healthcare system. **Cost/inconvenience** I’m concerned about the costs associated with the vaccine (such as costs associated with an office visit or the vaccine itself). It’s too inconvenient for me to get the vaccine (for example, due to the travel it would require or interference with my work hours). **Safety of COVID vaccine** I’m concerned that the vaccine was developed too quickly. I’m concerned about the side effects of the vaccine. I’m concerned about the long-term safety of the vaccine. I believe the vaccine could give me COVID-19. **Fear of vaccines in general** I don’t like needles. I don’t like vaccines in general.

Independent of vaccination status, concern over the safety of COVID-19 vaccines is the most cited reason for vaccine hesitancy (Fig 2). Many hesitant individuals perceive themselves as not being at high risk, while lack of trust features prominently among those refusing the vaccine. Among the vaccinated the most cited reasons for supporting vaccine uptake relate to positive externalities, namely their concern for others (79.7% compared with 24.7% of the unvaccinated, on average), while among the unvaccinated the most important reason is fear of COVID-19.

### Booster willingness among the vaccinated

**Fig 3:**
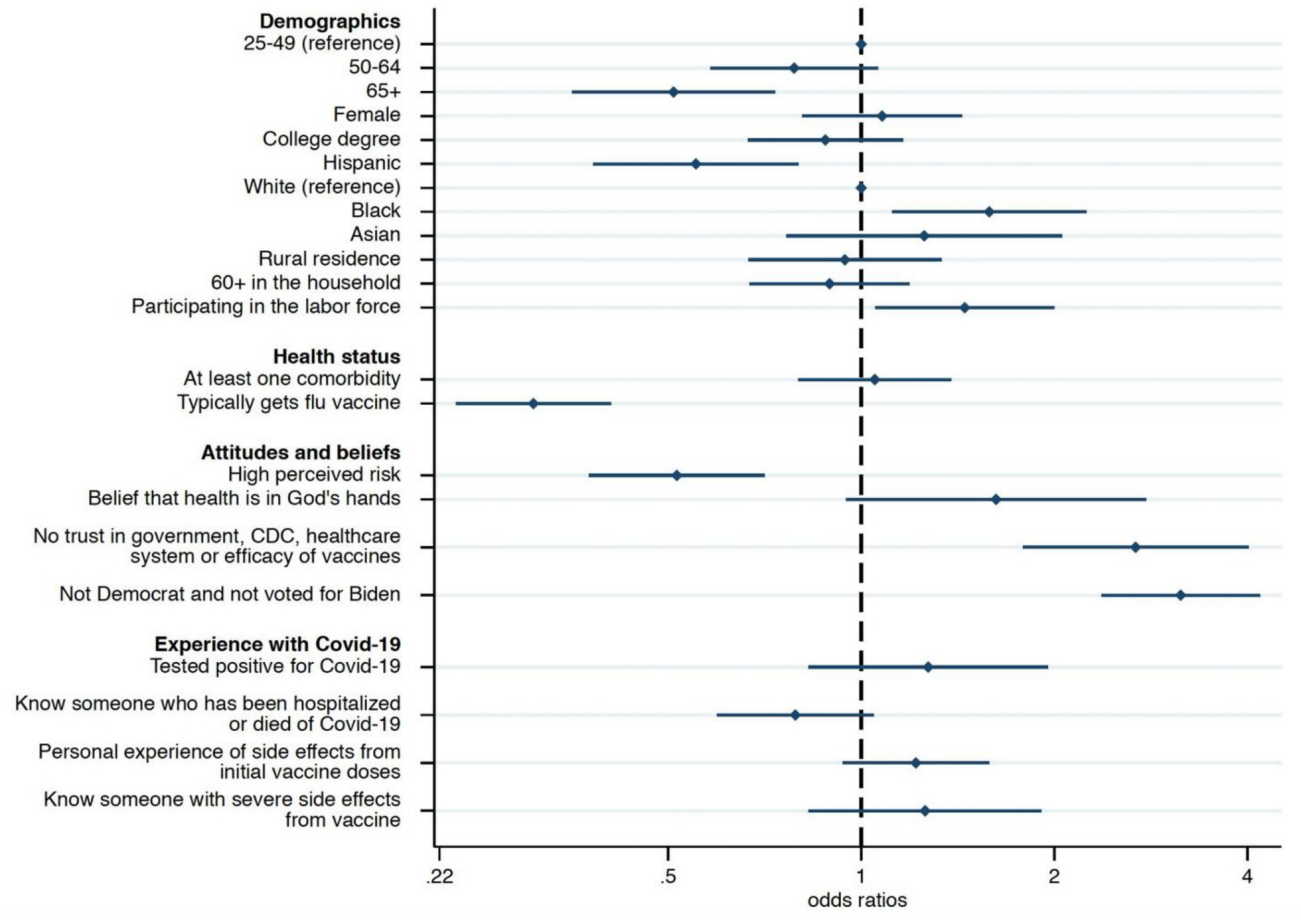
Factors associated with hesitancy/refusal to take the booster shot among fully vaccinated individuals aged 25 and older. *Notes*: Number of fully vaccinated individuals who are willing to take the booster shot = 1,280; number of fully vaccinated individuals who are hesitant or refuse the booster = 752. Odds ratios from logistic model predicting booster shot hesitancy/refusal among fully vaccinated individuals age 25 and older. The x-axis adopts a logarithmic scale. Weighted model. Inclusion of a dummy variable for respondents with missing income information (4.38% of the 25+ fully vaccinated sample has missing income information) and of regional and income covariates. Table 7 in S1 File shows regression results. The lines identify the 95% confidence intervals. Results are robust to the inclusion of the younger age group (18–24) and to the use of weights (Table 7 in S1 File).

Fig 3 reports the odds ratios (OR) from a logistic model predicting booster shot hesitancy or refusal conditional on being fully vaccinated. We find that many of the variables that exhibited a significant relationship with vaccine hesitancy or refusal also had such a relationship with the willingness to take a booster shot. In particular, lack of trust (OR: 2.85, 95% CI: 1.98–4.09, p<0.001), political affiliation (OR: 2.94, 95% CI: 2.28–3.81, p<0.001), flu vaccine habits (OR: 0.35, 95% CI: 0.27–0.45, p<0.001), and perception of being at high risk (OR: 0.50, 95% CI: 0.37–0.67, p<0.001) had the strongest associations with willingness to take the booster. Contrary to the results found regarding vaccine hesitancy or refusal, differences in educational attainment, rural residence, household composition, experience with COVID-19, and belief that health is in God’s hands no longer explain intentions. Presumably, this is because the sample of individuals asked about the booster shot is a select subset of the total sample who have already manifested their belief in vaccination.

### Incentives for vaccine adoption or disincentives for vaccine refusal

**Fig 4:**
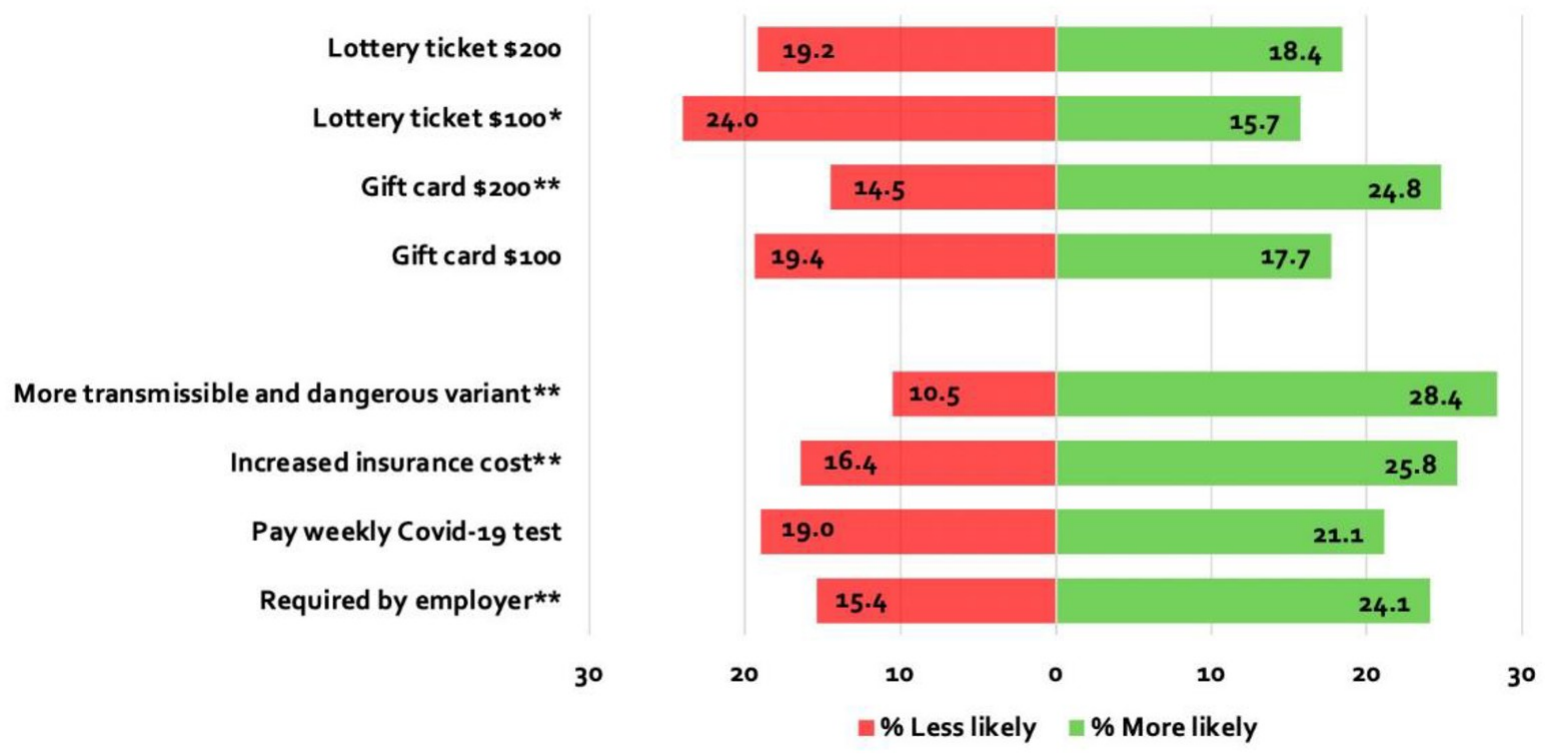
Change in willingness to be vaccinated compared with the baseline (%) among unvaccinated individuals. *Notes*: Each bar denotes the percentage of unvaccinated individuals who are more or less likely to get vaccinated in the incentive scenario compared with the baseline. To avoid response randomness, we excluded unvaccinated individuals who were in the bottom 10% and in the top 10% of the distribution of time to take the survey. That is, we excluded those who may not have taken the survey seriously enough (short duration) or who may have suffered from survey fatigue (long duration). Number of observations = 773. The values of the lottery ticket and of the gift card were randomly assigned across the respondents (although respondents were exposed to the same amount in the lottery question as in the gift card one). Number of observations with $100 gift card/lottery ticket = 385; number of observations with $200 gift card/lottery ticket = 388. Weighted results. Table 8 in S1 File shows figures. Table 9 in S1 File shows the results of t-tests that assess whether net movement (i.e., the proportion toward vaccination minus the proportion away from vaccination) differs significantly from zero. **: The significance test is accepted at the 1% level. *: The significant test is accepted at the 5% level.

Fig 4 illustrates the proportion of unvaccinated individuals who stated that incentives would increase, or even decrease, the likelihood that they would get vaccinated. Each measure seems to have a negative impact for some individuals, who state they are less likely to get vaccinated. We find that net movement—that is, the proportion toward vaccination minus the proportion away from vaccination—in response to monetary rewards is modest at best. Only the offer of a $200 gift card has a significant net impact on the willingness to vaccinate (10.3 percentage points, p = 0.005). In particular, as some of the literature already stresses, the prospect of winning a lottery has no substantial impact on intentions to get vaccinated [16]. If anything, the chance of winning the lottery might reduce the willingness to get vaccinated (net change with a $100 ticket is -8.3 percentage points, p-value = 0.02). Our result showing that a $200 gift is more effective than a gift of less value suggests the possibility that vaccine acceptance could increase further with greater monetary incentives.

In contrast, we find that the threat of immediate or potential harm results in a significant net increase in willingness to get vaccinated. The prospect of a more transmissible and dangerous variant increases willingness to get vaccinated by 17.9 percentage points (p<0.001). An employer vaccine mandate leads to a net increase in the probability of vaccine uptake of 8.7 percentage points (p<0.001), and a hypothetical $200 per month increase in health insurance premium increases the willingness by 9.4 percentage points (p<0.001), while a $10 per week payment for COVID-19 tests did not have a significant net effect on the probability of vaccine acceptance. These last two findings are consistent given that the added financial burden imposed by the higher insurance premium is several times greater than that associated with weekly tests.

Overall, these results conform with a long line of existing literature that has established that motivation stemming from monetary losses is greater than that generated from comparable gains [13]. This phenomenon, loss aversion, implies, for example, that the reduction in well-being from losing $10 is larger than the gain in well-being from winning $10. Thus, threatening to impose a penalty for nonvaccination is more effective than offering incentives for vaccination.

## Discussion and conclusions

As the COVID-19 pandemic proceeds, the United States and other nations continue to seek a path to its end. Yet, vaccine hesitancy and refusal hinder progress. This research attempts to understand who among us is more or less inclined to vaccinate, to intend to vaccinate if not having done so already, and willing to consider receiving a booster shot if already vaccinated. Further, we explore the reasons behind both vaccine acceptance and vaccine hesitancy. Last, to find a path forward, we examine scenarios that present either incentives or disincentives to vaccine uptake and judge their relative efficacy.

Among those who currently are hesitant or refuse to vaccinate, a core exists who will not be swayed, regardless of incentives or disincentives. Our analysis reveals that monetary rewards are perhaps less effective than other incentives, either due to the nature of the incentive itself or to the level of remuneration. At least in our scenarios, we find that lotteries, with their inherent uncertainty, are less effective than outright gifts. That said, our findings do indicate that money can be effective at some point. The question, then, revolves around the practical and moral issues that are bound to such incentives.

An alternative approach is to impose costs upon those who are hesitant or refuse to vaccinate. We find that the stick is generally more effective than the carrot. The costs that our scenarios posited would be quite substantial, resulting, for example, in job loss or markedly higher health insurance premiums. An employer vaccine mandate and an employer requirement that employees must pay sufficiently higher insurance premiums to absorb the increased healthcare costs not only for themselves but also for their work colleagues that would result from their lack of vaccination were found to significantly move respondents toward vaccination.

In our study, a significant proportion of unvaccinated individuals see benefits to vaccination (Fig 2). Almost 40% of those who are uncertain cite a concern for others as a reason to consider getting vaccinated. Fully half of those hesitant still express fear of contracting COVID-19 and thus have not yet closed the door to vaccination. As reasons for rejecting the vaccine, roughly 70% of the unvaccinated are concerned about the safety of COVID-19 vaccines and nearly 30% fear vaccines in general. These findings suggest an opening for improved vaccine uptake and argue for better targeted messaging from those in positions of authority and who interface with the public by virtue of their role, whether medical and public health personnel or government officials at the federal, state, and local levels. That said, the role of peers must be acknowledged, recognizing the need for messengers who are deemed most worthy of trust by those who have yet to make a decision [17].

Also, worth exploring further is the tight association between vaccine uptake for the flu and for COVID-19. This relationship raises the intriguing question of whether engaging in a substantial public health campaign to build confidence in the safety and efficacy of flu vaccines would be worthwhile. With that greater confidence in place, the road to vaccine uptake in the next, if not in the current, pandemic may have one less significant obstacle to hinder its success.

Our analysis relies on data collected through an online survey administered to a nonprobability opt-in survey panel. Both the online mode and the sampling frame, at least theoretically, raise selection-bias concerns. Although the sample is demographically representative of the U.S. population, it may not be strictly representative of attitudes towards COVID-19 and COVID-19 vaccination. For example, those who are more skeptical about vaccines may be less inclined to participate in a survey about COVID-19 vaccination. Similarly, those who are most negatively affected by COVID-19 may not have the time or the resources to participate in the survey. Other limitations reflect the common issue of reliance upon self-reported data, including those that assess prior COVID-19 infection and intentions to get vaccinated.

This research contributes to our understanding of how to better motivate the unvaccinated. However, given current levels of vaccination, clearly more work must be done to identify ways that resonate with the unvaccinated that would reveal how vaccination is in their self-interest or to ascertain means that will surface their desire to help those other than themselves.

## Supporting information

S1 File Supplementary material. (DOCX)

S2 File Instrument. (DOCX)
