# Supplementary material for: Factors underlying COVID-19 vaccine and booster hesitancy and refusal, and incentivizing vaccine adoption
Source: PLoS One. 2022 Sep 22;17(9):e0274529. doi: 10.1371/journal.pone.0274529 (PMC9498968; doi:10.1371/journal.pone.0274529)
Supplement: S1 File — (DOCX) [file pone.0274529.s001.docx]

**Factors Underlying Covid-19 Vaccine and Booster Hesitancy and Refusal, And Incentivizing Vaccine Adoption**

**Supplementary information**

Neil G. Bennett PhD, David E. Bloom PhD, and Maddalena Ferranna PhD

**eTable 1. Distribution of survey respondents compared to U.S. population (American Community Survey 2015-2019)**

|  | **ACS (18+) %** | **Survey**  **Unweighted Number (%)** | **Survey**  **Weighted %** |
| --- | --- | --- | --- |
| Gender  Male  Female | 48.7  51.3 | 1,697 (48.5)  1,800 (51.5) | 48.7  51.3 |
| Age group  18-24  25-49  50-64  65+ | 12.2  42.5  25.1  20.2 | 396 (11.3)  1,595 (45.6)  747 (21.4)  759 (21.7) | 12.2  42.5  25.1  20.2 |
| Race/ethnic group  Hispanic  Non-Hispanic White  Non-Hispanic Black  Other | 16.0  63.6  12.0  8.4 | 739 (21.1)  1,705 (48.8)  760 (21.7)  293 (8.4) | 15.9  63.6  12.0  8.5 |
| Education  High school or less  Some college  College or more | 39.7  30.8  29.5 | 1,312 (37.5)  1,010 (28.9)  1,175 (33.6) | 39.7  30.8  29.5 |

*Notes:* Survey weights are computed using the raking method (svycal rake command in Stata) based on the gender-specific marginal distributions of education, age and race/ethnicity displayed in eTable 2. Data from the American Community Survey (ACS) 2015-2019 are weighted.

**eTable 2. Distribution of survey respondents by gender compared to the U.S. population (American Community Survey 2015-2019)**

|  | **Male** | | | **Female** | | |
| --- | --- | --- | --- | --- | --- | --- |
|  | **ACS %** | **Survey**  **Unweighted Number (%)** | **Survey Weighted %** | **ACS %** | **Survey**  **Unweighted Number (%)** | **Survey Weighted %** |
| Age group  18-24  25-49  50-64  65+ | 12.8  43.7  25.1  18.4 | 136 (8.0)  849 (50.0)  374 (22.1)  338 (19.9) | 12.8  43.7  25.2  21.9 | 11.6  41.3  25.2  21.9 | 260 (14.5)  746 (41.4)  373 (20.7)  421 (23.4) | 11.6  41.3  25.2  21.9 |
| Race/ethnic group  Hispanic  Non-Hispanic White  Non-Hispanic Black  Other | 16.5  63.8  11.5  8.2 | 251 (14.8)  1,048 (61.8)  267 (15.7)  131(7.7) | 16.5  63.8  11.5  8.2 | 15.4  63.5  12.4  8.7 | 488 (27.1)  657 (36.5)  493 (27.4)  162 (9.0) | 15.4  63.5  12.4  8.7 |
| Education  High school or less  Some college  College or more | 41.8  29.6  28.6 | 486 (28.6)  502 (29.6)  709 (41.8) | 41.8  29.6  28.6 | 37.7  32.0  30.3 | 826 (45.9)  508 (28.2)  466 (25.9) | 37.7  32.0  30.3 |

*Notes:* Survey weights are computed using the raking method (svycal rake command in Stata) based on the gender-specific marginal distributions of education, age and race/ethnicity displayed in eTable 2. Data from the American Community Survey (ACS) 2015-2019 are weighted.

**eTable 3. Vaccination status by sociodemographic group**

|  | **Fully vaccinated** | **Partially vaccinated** | **Undecided** | **Unwilling** |
| --- | --- | --- | --- | --- |
| **Total** | 61.5 (59.6-63.4) | 6.1 (5.3-7.0) | 18.3 (16.9-19.8) | 14.1 (12.8-15.6) |
| **Gender**  Male  Female | 61.7 (59.1-64.2)  61.3 (58.6-64.0) | 7.1 (5.9-8.6)  5.1 (4.1-6.4) | 18.6 (16.6-20.7)  18.0 (16.0-20.3) | 12.6 (10.9-14.6)  15.5 (13.5-17.8) |
| **Age group**  18-24  25-49  50-64  65+ | 42.2 (36.6-48.0)  50.9 (48.1-53.7)  69.0 (65.0-72.7)  86.1 (83.1-88.7) | 10.2 (7.1-14.4)  7.5 (6.2-9.1)  4.1 (2.9-6.0)  3.0 (1.9-4.7) | 27.9 (23.0-33.2)  24.8 (22.5-27.3)  13.1 (10.5-16.2)  5.3 (3.7-7.4) | 19.7 (15.5-24.8)  16.8 (14.7-19.1)  13.7 (11.0-17.0)  5.6 (3.9-7.9) |
| **Education**  High school or less  Some college  College or more | 45.7 (42.7-48.8)  62.6 (59.1-66.0)  81.2 (78.8-84.1) | 6.8 (5.5-8.3)  5.4 (4.1-7.2)  5.8 (4.5-7.4) | 27.1 (24.5-29.9)  17.1 (14.6-20.0)  7.6 (6.0-9.6) | 20.3 (17.9-22.9)  14.8 (12.3-17.7)  5.0 (3.6-6.9) |
| **Race/ethnicity**  Non-Hispanic White  Hispanic  Non-Hispanic Black  Asian  Other | 59.6 (57.0-62.2)  61.2 (57.1-65.2)  56.5 (52.6-60.4)  89.5 (84.2-93.1)  65.9 (53.4-76.3) | 4.5 (3.6-5.7)  10.0 (7.7-12.8)  10.3 (8.1-13.0)  5.0 (2.6-9.5)  3.5 (1.2-9.5) | 18.8 (16.9-20.9)  20.3 (17.1-23.9)  21.6 (18.5-25.1)  3.9 (2.0-7.6)  10.9 (5.9-19.2) | 17.0 (15.1-19.1)  8.5 (6.4-11.3)  11.6 (9.3-14.4)  1.6 (0.5-4.8)  19.7 (11.2-32.3) |
| **Region**  North-East  Mid-West  South  West | 70.6 (66.3-74.6)  57.1 (53.1-61.1)  58.4 (55.2-61.5)  63.7 (59.8-67.4) | 5.9 (4.2-8.2)  6.3 (4.5-8.6)  6.0 (4.7-7.5)  6.3 (4.7-8.5) | 13.5 (10.8-16.9)  20.6 (17.5-24.0)  20.4 (18.0-23.0)  16.4 (13.6-19.6) | 10.0 (7.4-13.3)  16.1 (13.3-19.3)  15.3 (13.0-17.9)  13.6 (11.0-16.7) |
| **Residence**  Urban  Suburban  Rural | 60.4 (57.1-63.5)  69.1 (66.2-71.8)  52.1 (47.9-56.3) | 9.7 (7.9-11.8)  5.0 (3.9-6.4)  3.5 (2.3-5.4) | 19.3 (16.8-22.0)  15.8 (13.7-18.1)  20.3 (17.3-23.8) | 10.6 (8.7-12.9)  10.1 (8.4-12.1)  24.0 (20.5-27.9) |
| **Political affiliation**  Democrat  Independent  Republican | 74.6 (72.0-77.1)  55.9 (52.1-59.6)  56.3 (52.4-60.2) | 7.3 (5.9-9.0)  5.6 (5.2-7.5)  4.7 (3.3-9.9) | 13.2 (11.3-15.3)  21.4 (18.5-24.6)  18.5 (15.6-21.7) | 4.9 (3.8-6.3)  17.1 (14.3-20.3)  20.5 (17.4-24.0) |
| **Vote in 2020 election**  Biden  Trump  Didn’t vote | 78.5 (76.1-80.7)  53.0 (49.3-56.6)  42.7 (38.6-46.9) | 6.5 (5.3-8.0)  6.2 (4.7-8.2)  3.8 (6.7-15.9) | 11.0 (9.4-12.9)  19.8 (17.1-22.9)  28.9 (25.3-32.7) | 4.0 (3.0-5.2)  21.0 (18.1-24.2)  24.6 (21.1-28.4) |
| **Religion**  Evangelical Christian  Non-Evangelical Christian  Jewish  Muslim  No religion | 60.1 (56.1-64.0)  71.7 (67.7-75.4)  77.6 (64.8-86.7)  63.9 (49.2-76.4)  59.3 (55.6-62.9) | 8.5 (6.6-10.9)  3.1 (2.1-4.6)  8.9 (3.4-21.4)  16.8 (8.6-30.5)  5.1 (3.7-6.9) | 18.8 (15.9-22.2)  14.7 (11.9-17.9)  6.8 (2.9-14.9)  7.7 (3.1-18.2)  19.8 (17.0-22.9) | 12.5 (10.0-15.6)  10.5 (8.0-13.8)  6.8 (2.4-17.8)  11.5 (4.3-27.5)  15.8 (13.2-18.8) |
| **Comorbidities**  At least one  No comorbidity | 66.2 (63.6-68.7)  56.1 (53.3-58.9) | 6.3 (5.2-7.6)  5.9 (4.8-7.3) | 15.9 (14.0-18.0)  21.0 (18.9-23.3) | 11.6 (9.9-13.5)  17.0 (14.9-19.3) |
| **Flu vaccine**  Take flu vaccine  Don’t take flu vaccine | 79.5 (77.3-81.5)  41.4 (38.7-44.2) | 7.1 (5.9-8.5)  5.0 (3.9-6.3) | 10.2 (8.8-11.9)  27.3 (24.8-29.8) | 3.1 (2.3-4.3)  26.4 (23.9-29.0) |
| **Income**  Less than 50k  50-100k  More than 100k | 54.8 (52.1-57.4)  67.9 (64.2-71.3)  77.1 (72.7-80.9) | 5.8 (4.8-7.0)  6.2 (4.6-8.3)  7.3 (5.0-10.6) | 21.7 (19.7-24.0)  14.8 (12.3-17.6)  10.7 (8.1-14.0) | 17.7 (15.7-19.9)  11.2 (8.9-13.9)  4.9 (3.2-7.5) |

*Notes:*  95% logit-transformed confidence intervals in parentheses.

**eTable 4. Willingness to take the booster shot among the fully vaccinated by sociodemographic group**

|  | **Willing** | **Undecided** | **Unwilling** |
| --- | --- | --- | --- |
| **Total** | 60.2 (57.8-62.5) | 36.3 (34.1-38.7) | 3.4 (2.6-4.5) |
| **Gender**  Male  Female | 59.5 (56.3-62.5)  60.9 (57.3-64.4) | 37.8 (34.7-40.9)  35.0 (31.6-38.5) | 2.8 (1.9-4.1)  4.1 (2.8-5.9) |
| **Age group**  18-24  25-49  50-64  65+ | 35.4 (27.4-44.4)  51.9 (48.0-55.8)  61.8 (57.0-66.4)  76.3 (72.4-80.0) | 58.2 (49.3-66.7)  43.3 (39.5-47.2)  35.4 (31.0-40.2)  22.1 (18.7-25.9) | 6.3 (2.2-11.9)  4.8 (3.3-6.9)  2.8 (1.5-5.2)  2.6 (0.7-3.5) |
| **Education**  High school or less  Some college  College or more | 51.8 (47.3-56.3)  60.8 (56.4-65.0)  66.1 (62.4-69.6) | 43.7 (39.3-48.2)  35.8 (31.7-40.1)  31.2 (27.9-34.8) | 4.5 (3.0-6.9)  3.4 (2.1-5.5)  2.6 (1.5-4.4) |
| **Race/ethnicity**  Non-Hispanic White  Hispanic  Non-Hispanic Black  Asian  Other | 59.8 (56.5-63.0)  63.2 (58.1-68.0)  59.7 (54.7-64.6)  60.9 (53.5-67.9)  50.4 (36.1-64.6) | 36.4 (33.2-39.6)  32.9 (28.3-37.9)  38.7 (33.9-43.8)  38.1 (31.2-45.4)  41.2 (27.8-56.1) | 3.8 (2.7-5.5)  3.9 (2.5-6.1)  1.5 (0.7-3.2)  1.0 (0.1-6.6)  8.4 (3.1-20.9) |
| **Region**  North-East  Mid-West  South  West | 61.6 (56.3-66.7)  56.4 (51.1-61.6)  57.3 (53.1-61.3)  66.7 (62.1-71.0) | 35.1 (30.2-40.3)  39.5 (34.4-44.7)  38.8 (34.8-43.0)  31.0 (26.8-35.6) | 3.3 (1.6-6.5)  4.1 (2.4-7.1)  3.9 (2.7-5.8)  2.3 (1.1-4.7) |
| **Residence**  Urban  Suburban  Rural | 62.3 (58.2-66.2)  61.1 (57.6-64.4)  57.1 (51.3-62.7) | 34.0 (30.1-38.0)  35.9 (32.7-39.3)  38.5 (33.1-44.2) | 3.7 (2.4-5.8)  3.0 (1.9-4.6)  4.4 (2.5-7.6) |
| **Political affiliation**  Democrat  Independent  Republican | 71.7 (68.5-74.7)  52.1 (47.2-57.0)  52.2 (47.0-57.3) | 25.8 (22.9-29.0)  44.4 (39.6-49.3)  41.7 (36.7-46.8) | 2.5 (1.5-4.0)  3.5 (2.1-5.7)  6.1 (3.9-9.5) |
| **Vote in 2020 election**  Biden  Trump  Didn’t vote | 73.0 (70.1-75.7)  48.4 (43.5-53.2)  44.7 (38.4-51.1) | 25.4 (22.8-28.2)  44.1 (39.4-49.0)  52.1 (45.6-58.4) | 1.6 (0.9-2.8)  7.5 (5.2-10.7)  3.2 (1.6-6.3) |
| **Religion**  Evangelical Christian  Non-Evangelical Christian  Jewish  Muslim  No religion | 57.8 (52.7-62.8)  70.6 (65.9-74.9)  71.8 (58.2-82.3)  67.5 (51.4-80.3)  58.9 (54.2-63.5) | 38.9 (34.0-44.0)  27.4 (23.3-32.0)  25.5 (15.4-38.9)  28.0 (16.2-43.8)  35.9 (31.5-40.5) | 3.3 (1.9-5.7)  2.0 (1.0-4.0)  2.8 (0.7-10.4)  4.5 (1.0-17.8)  5.2 (3.2-8.2) |
| **Comorbidities**  At least one  No comorbidity | 65.5 (62.4-68.4)  53.2 (49.4-56.9) | 31.7 (28.8-34.7)  42.5 (38.9-46.2) | 2.8 (1.9-4.1)  4.3 (2.9-6.3) |
| **Flu vaccine**  Take flu vaccine  Don’t take flu vaccine | 69.4 (66.6-72.0)  40.6 (36.4-45.0) | 28.9 (26.3-31.6)  52.3 (48.0-56.6) | 1.8 (1.1-2.7)  7.1 (5.0-9.9) |
| **Income**  Less than 50k  50-100k  More than 100k | 58.5 (54.9-62.0)  62.2 (57.9-66.4)  65.9 (60.7-70.8) | 37.3 (33.9-40.8)  34.3 (30.3-38.5)  32.0 (27.2-37.1) | 4.2 (2.9-6.1)  3.5 (2.1-5.7)  2.2 (1.1-4.2) |

*Notes:*  95% logit-transformed confidence intervals in parentheses.

**eTable 5. Relative risk ratios from multinomial logistic regression model predicting vaccine hesitancy (“undecided”) and refusal (“unwilling”) relative to those partially or fully vaccinated.**

| **Characteristics** | **(1)**  **25+ sample** | | **(2)**  **Entire sample** | | **(3)**  **25+ sample, design weights** | |
| --- | --- | --- | --- | --- | --- | --- |
|  | **Undecided** | **Unwilling** | **Undecided** | **Unwilling** | **Undecided** | **Unwilling** |
| ***Socio-demographic characteristics*** |  |  |  |  |  |  |
| **Age group**  18-24 | - | - | - | - | - | - |
| 25-49 | - | - | 0.94  (0.67-1.32) | 0.84  (0.53-1.32) | - | - |
| 50-64 | 0.53 ***  (0.38-0.73) | 0.72  (0.48-1.08) | 0.50***  (0.33-0.76) | 0.61*  (0.36-1.03) | 0.49***  (0.36-0.65) | 0.72*  (0.50-1.05) |
| 65+ | 0.30***  (0.19-0.47) | 0.50**  (0.27-0.93) | 0.28***  (0.17-0.48) | 0.40***  (0.20-0.79) | 0.31***  (0.21-0.47) | 0.46***  (0.26-0.79) |
| **Female** | 1.11  (0.84-1.45) | 1.56**  (1.08-2.25) | 1.18  (0.92-1.50) | 1.39*  (0.99-1.94) | 1.15  (0.89-1.47) | 1.60**  (1.12-2.29) |
| **College** | 0.32***  (0.23-0.45) | 0.31***  (0.19-0.51) | 0.35***  (0.26-0.49) | 0.33***  (0.21-0.53) | 0.37***  (0.28-0.50) | 0.34***  (0.22-0.51) |
| **Hispanic** | 0.98  (0.70-1.37) | 0.49***  (0.30-0.78) | 0.99  (0.74-1.32) | 0.54***  (0.35-0.84) | 0.88  (0.65-1.20) | 0.48***  (0.31-0.74) |
| **Race**  White (Ref.) |  |  |  |  |  |  |
| Black | 1.64***  (1.18-2.27) | 1.89***  (1.20-2.99) | 1.68***  (1.25-2.27) | 2.00***  (1.32-3.03) | 1.51***  (1.12-2.04) | 1.62**  (1.05-2.50) |
| Asian | 0.35***  (0.16-0.73) | 0.17**  (0.04-0.66) | 0.29***  (0.14-0.62) | 0.17***  (0.04-0.63) | 0.33***  (0.16-0.67) | 0.16***  (0.04-0.59) |
| Other | 0.88  (0.50-1.56) | 2.45***  (1.24-4.83) | 0.98  (0.59-1.63) | 2.33***  (1.26-4.30) | 0.99  (0.57-1.71) | 3.06***  (1.64-5.70) |
| **Rural residence** | 1.12  (0.82-1.53) | 1.60**  (1.09-2.36) | 1.16  (0.87-1.54) | 1.79***  (1.25-2.57) | 1.13  (0.85-1.50) | 1.53**  (1.08-2.16) |
| **Region**  North-East |  | - |  |  |  |  |
| Mid-West | 1.42  (0.93-2.17) | 0.99  (0.57-1.70) | 1.28  (0.87-1.88) | 1.23  (0.74-2.04) | 1.22  (0.84-1.77) | 0.97  (0.59-1.60) |
| South | 1.57**  (1.05-2.36) | 1.30  (0.77-2.20) | 1.36  (0.94-1.96) | 1.39  (0.86-2.27) | 1.46**  (1.03-2.08) | 1.18  (0.74-1.89) |
| West | 1.52*  (0.98-2.34) | 1.71*  (0.97-3.04) | 1.48*  (1.00-2.19) | 1.94**  (1.14-3.31) | 1.37  (0.93-2.02) | 1.58*  (0.95-2.62) |
| **60+ in the household** | 0.81  (0.60-1.09) | 0.53***  (0.34-0.82) | 0.76*  (0.57-1.01) | 0.53***  (0.35-0.79) | 0.78*  (0.60-1.03) | 0.55***  (0.37-0.81) |
| **Participating in the labor force** | 1.24  (0.89-1.71) | 0.77  (0.52-1.16) | 1.27  (0.94-1.72) | 0.70*  (0.49-1.02) | 1.18  (0.87-1.58) | 0.76  (0.52-1.09) |
| **Low income** | 1.09  (0.83-1.45) | 1.29  (0.88-1.88) | 1.24  (0.95-1.61) | 1.33  (0.93-1.90) | 1.12  (0.87-1.44) | 1.45**  (1.02-2.05) |
| **No income** | 1.33  (0.78-2.28) | 0.91  (0.41-2.04) | 1.20  (0.73-1.95) | 1.28  (0.63-2.60) | 1.25  (0.76-2.06) | 0.93  (0.43-2.02) |
| ***Health status*** |  |  |  |  |  |  |
| **At least one comorbidity** | 1.10  (0.84-1.43) | 0.92  (0.65-1.29) | 1.06  (0.83-1.35) | 0.98  (0.71-1.34_ | 1.01  (0.79-1.29) | 0.87  (0.64-1.19) |
| **Typically gets flu vaccine** | 0.28***  (0.21-0.36) | 0.08***  (0.05-0.13) | 0.28***  (0.22-0.36) | 0.09***  (0.06-0.13) | 0.28***  (0.22-0.36) | 0.09***  (0.06-0.13) |
| ***Attitudes and beliefs*** |  |  |  |  |  |  |
| **High perceived risk** | 0.90  (0.64-1.26) | 0.72  (0.45-1.16) | 0.87  (0.64-1.19) | 0.82  (0.52-1.27) | 0.77*  (0.57-1.04) | 0.74  (0.48-1.13) |
| **Belief that health is in God’s hands** | 2.05***  (1.45-2.91) | 2.54***  (1.67-3.88) | 1.93***  (1.39-2.69) | 2.54***  (1.70-3.80) | 2.04***  (1.49-2.80) | 2.33***  (1.60-3.40) |
| **No trust in government, CDC, healthcare system or efficacy of vaccines** | 2.86***  (2.13-3.83) | 9.07***  (6.40-12.86) | 2.80***  (2.13-3.66) | 8.33***  (6.05-11.45) | 2.45***  (1.87-3.21) | 8.54***  (6.22-11.74) |
| **Not Democrat and not voted for Biden** | 2.27***  (1.71-3.00) | 4.42***  (2.87-6.77) | 2.31***  (1.79-2.98) | 4.57***  (3.14-6.67) | 2.20***  (1.71-2.82) | 4.29***  (2.93-6.29) |
| ***Experience with COVID-19*** |  |  |  |  |  |  |
| **Know someone with severe side effects from vaccine** | 0.86  (0.66-1.14) | 2.68***  (1.79-4.01) | 0.81  (0.63-1.04) | 2.40***  (1.66-3.46) | 1.14  (0.83-1.56) | 2.45***  (1.69-3.56) |
| **Tested positive for Covid-19** | 1.30  (0.91-1.85) | 1.07  (0.62-1.86) | 1.09  (0.63-1.04) | 1.02  (0.62-1.67) | 1.27  (0.92-1.76) | 0.95  (0.59-1.54) |
| **Know someone who has been hospitalized or died of Covid-19** | 1.20  (0.85-1.69) | 0.58***  (0.40-0.84) | 1.23  (0.90-1.69) | 0.58***  (0.42-0.82) | 0.78**  (0.60-1.00) | 0.59***  (0.42-0.83) |
| Constant | 0.21 | 0.07 | 0.22 | 0.08 | 0.28 | 0.08 |
| Number of observations | 3,101 | | 3,497 | | 3,101 | |
| Pseudo R2 | 0.3169 | | 0.3072 | | 0.3079 | |

*Notes:* Overall, 2440 individuals are partially or fully vaccinated, 635 are undecided, and 422 are unwilling to get vaccinated. Among individuals aged 25 and over (25+ sample), 2,232 are partially or fully vaccinated, 350 are unwilling to get vaccinated, and 519 are undecided.

Specifications (1) and (2) use the weights based on gender-specific distributions of education, age and race/ethnic group as in eTable 1 and 2. Specification (3) uses design weights that account for the oversampling of racial and ethnic minorities.

The constant estimates baseline relative risk ratio for each outcome.

95% confidence intervals in parentheses. Significance levels: *** p<0.01; ** p<0.05; * p<0.1.

**eTable 6. Percentage of respondents selecting a particular reason for accepting/refusing the COVID-19 vaccine, by vaccination status.**

| **Reason** | **Total** | **Fully vaccinated** | **Partially vaccinated** | **Undecided** | **Unwilling** |
| --- | --- | --- | --- | --- | --- |
| **Safety of COVID-19 vaccines** | 42.0  (40.1-43.9) | 26.3  (24.2-28.4) | 45.3  (38.1-52.7) | 70.8  (66.7-74.6) | 71.7  (66.7-76.2) |
| **No need** | 31.3  (29.6-33.1) | 18.5  (16.8-20.4) | 38.3  (31.4-45.6) | 51.3  (46.9-55.7) | 58.0  (52.6-63.2) |
| **Lack of trust** | 24.7  (23.1-26.4) | 12.1  (10.6-13.7) | 19.6  (14.5-26.0) | 37.1  (32.9-41.6) | 65.9  (60.6-70.8) |
| **Fear of vaccines in general** | 17.0  (15.6-18.5) | 10.3  (9.0-11.8) | 18.0  (12.8-24.6) | 29.5  (25.6-33.8) | 29.6  (25.0-34.7) |
| **Cost/inconvenience** | 7.6  (6.7-8.6) | 6.5  (5.5-7.7) | 18.9  (13.7-25.5) | 9.7  (7.5-12.6) | 4.4  (2.8-7.0) |
| **Fear of COVID-19** | 61.1  (59.2-63.0) | 74.3  (72.1-76.3) | 63.9  (56.6-70.8) | 51.0  (46.5-55.4) | 15.7  (12.3-19.8) |
| **Concern for others** | 61.9  (60.0-63.7) | 81.6  (79.6-83.4) | 60.5  (53.0-67.6) | 38.7  (34.5-43.1) | 6.5  (4.4-9.4) |
| **Requirement** | 28.4  (26.7-30.1) | 33.1  (30.9-35.4) | 36.4  (29.7-43.6) | 26.6  (22.9-30.7) | 6.7  (4.6-9.7) |

*Notes:* Weighted results. 95% logit-transformed confidence intervals in parentheses.

**eTable 7. Odds ratios from logistic regression model predicting booster shot hesitancy or refusal among fully vaccinated individuals.**

| **Characteristics** | **(1)**  **25+ sample** | **(2)**  **Full sample** | **(1)**  **25+ sample, design weight** |
| --- | --- | --- | --- |
| ***Socio-demographic characteristics*** |  |  |  |
| **Age group**  18-24 | - | - | - |
| 25-49 | - | 0.47***  (0.29-0.74) | - |
| 50-64 | 0.87  (0.64-1.18) | 0.40***  (0.24-0.66) | 0.85  (0.64-1.11) |
| 65+ | 0.60***  (0.42-0.87) | 0.27***  (0.16-0.47) | 0.58***  (0.42-0.80) |
| **Female** | 1.02  (0.79-1.32) | 1.00  (0.79-1.28) | 1.04  (0.82-1.33) |
| **College** | 0.81  (0.61-1.06) | 0.76**  (0.59-1.00) | 0.78**  (0.61-0.99) |
| **Hispanic** | 0.77  (0.57-1.05) | 0.77*  (0.57-1.05) | 0.77*  (0.57-1.03) |
| **Race**  White (Ref.) |  |  |  |
| Black | 1.55***  (1.12-2.14) | 1.55***  (1.14-2.11) | 1.58***  (1.17-2.13) |
| Asian | 1.20  (0.77-1.87) | 1.38  (0.91-2.10) | 1.20  (0.79-1.83) |
| Other | 1.50  (0.97-2.33) | 1.42  (0.91-2.20) | 1.58**  (1.03-2.42) |
| **Rural residence** | 0.92  (0.66-1.27) | 1.01  (0.74-1.38) | 0.93  (0.70-1.24) |
| **Region**  North-East (ref.) |  |  |  |
| Mid-West | 1.00  (0.70-1.45) | 0.99  (0.70-1.41) | 1.12  (0.80-1.57) |
| South | 1.02  (0.73-1.43) | 1.01  (0.73-1.40) | 1.04  (0.76-1.42) |
| West | 0.78  (0.55-1.11) | 0.76  (0.54-1.07) | 0.80  (0.58-1.11) |
| **60+ in the household** | 0.79*  (0.61-1.03) | 0.76**  (0.59-0.97) | 0.74**  (0.58-0.94) |
| **Participating in the labor force** | 1.49**  (1.10-2.02) | 1.39**  (1.04-1.86) | 1.41**  (1.08-1.85) |
| **Low income** | 1.03  (0.79-1.35) | 1.02  (0.79-1.32) | 1.00  (0.79-1.28) |
| **No income** | 2.02**  (1.16-3.52) | 1.91**  (1.14-3.18) | 2.10***  (1.29-3.44) |
| ***Health status*** |  |  |  |
| **At least one comorbidity** | 0.98  (0.76-1.26) | 0.94  (0.74-1.20) | 0.92  (0.73-1.15) |
| **Typically gets flu vaccine** | 0.35***  (0.27-0.45) | 0.38***  (0.29-0.48) | 0.33***  (0.26-0.41) |
| ***Attitudes and beliefs*** |  |  |  |
| **High perceived risk** | 0.50***  (0.37-0.67) | 0.45***  (0.34-0.60) | 0.56***  (0.43-0.72) |
| **Belief that health is in God’s hands** | 1.30  (0.81-2.09) | 1.22  (0.77-1.95) | 1.24  (0.82-1.87) |
| **No trust in government, CDC, healthcare system or efficacy of vaccines** | 2.85***  (1.98-4.09) | 2.72***  (1.90-3.89) | 2.81***  (2.02-3.91) |
| **Not Democrat and not voted for Biden** | 2.94***  (2.28-3.81) | 2.92***  (2.28-3.75) | 3.09***  (2.45-3.90) |
| ***Experience with COVID-19*** |  |  |  |
| **Know someone with severe side effects from vaccine** | 1.20  (0.82-1.75) | 1.18  (0.82-1.70) | 1.17  (0.84-1.62) |
| **Personal experience of side effects from vaccine** | 1.13  (0.89-1.45) | 1.08  (0.85-1.36) | 1.21*  (0.97-1.51) |
| **Tested positive for Covid-19** | 1.28  (0.87-1.89) | 1.40*  (0.98-2.00) | 1.12  (0.80-1.57) |
| **Know someone who has been hospitalized or died of Covid-19** | 0.78*  (0.60-1.01) | 0.75**  (0.59-0.97) | 0.81*  (0.64-1.02) |
| Constant | 0.80 | 1.95 | 0.87 |
| Number of observations | 2,032 | 2,200 | 2,032 |
| Pseudo R2 | 0.1831 | 0.1916 | 0.1890 |

*Notes:* Overall, 1,338 fully vaccinated individuals are willing to take the booster shot, and 862 are undecided. Among individuals aged 25 and over (25+ sample), 1,280 are willing to take the booster shot, and 752 are hesitant or refuse the booster. Specifications (1) and (2) use the weights based on gender-specific distributions of education, age and race/ethnic group as in eTable 2. Specification (3) uses design weights that account for the oversampling of racial and ethnic minorities.

The constant estimates baseline odds.

95% confidence intervals in parentheses. Significance levels: *** p<0.01; ** p<0.05; * p<0.1.

**eTable 8. Percentage of the unvaccinated that is more or less likely to get vaccinated, by incentive/disincentive scenario.**

| **Scenario** | **More likely** | **As likely as before** | **Less likely** |
| --- | --- | --- | --- |
| **Lottery ticket $100** | 15.7  (12.1-20.2) | 60.3  (54.7-65.7) | 24.0  (19.6-29.0) |
| **Lottery ticket $200** | 18.4  (14.4-23.3) | 62.4  (56.9-67.7) | 19.2  (15.3-23.7) |
| **Gift card $100** | 17.7  (14.0-22.2) | 62.9  (57.4-68.1) | 19.4  (15.4-24.1) |
| **Gift card $200** | 24.8  (20.1-30.1) | 60.7  (55.1-66.1) | 14.5  (11.1-18.8) |
| **Required by employer** | 24.1  (20.8-27.7) | 60.5  (56.5-64.3) | 15.4  (12.8-18.4) |
| **Pay weekly COVID-19 test** | 21.1  (18.0-24.5) | 59.9  (56.0-63.8) | 19.0  (16.1-22.2) |
| **Increased insurance cost** | 25.8  (22.5-29.5) | 57.8  (53.8-61.6) | 16.4  (13.7-19.5) |
| **More transmissible and dangerous variant** | 28.4  (24.9-32.1) | 61.1  (57.1-64.9) | 10.5  (8.4-13.1) |

*Notes:* To avoid response randomness, we excluded non-vaccinated individuals who were in the bottom 10% and in the top 10% of the distribution of time to take the survey. That is, we exclude those who may not have taken the survey seriously enough (short duration) or who may suffer from survey fatigue (long duration). Number of observations = 773. The values of the lottery ticket and of the gift card were randomly assigned across the respondents (although respondents were exposed to the same amount in the lottery question as in the gift card one). Number of observations with $100 gift card/lottery ticket = 385; number of observations with $200 gift card/lottery ticket = 388.

“More likely” denotes the percentage of non-vaccinated respondents who select a higher value in the intention to get vaccinated scale (1= the respondent will definitely get vaccinated to 5= the respondent will definitely not get vaccinated) under the incentive/disincentive scenario compared to the baseline.

“Less likely” denotes the percentage of non-vaccinated respondents who select a lower value in the intention to get vaccinated scale (1= the respondent will definitely get vaccinated to 5= the respondent will definitely not get vaccinated) under the incentive/disincentive scenario compared to the baseline.

“As likely as before” denotes the percentage of non-vaccinated respondents who select the same value in the intention to get vaccinated scale (1= the respondent will definitely get vaccinated to 5= the respondent will definitely not get vaccinated) under the incentive/disincentive scenario compared to the baseline.

Weighted results. 95% logit-transformed confidence intervals in parentheses.

**eTable 9. Results of testing the hypothesis that the average changes in willingness to get vaccinated is zero.**

| **Scenario** | **t-statistic** | **p-value** |
| --- | --- | --- |
| **Lottery ticket $100** | -2.38 | 0.0179 |
| **Lottery ticket $200** | -0.21 | 0.8326 |
| **Gift card $100** | -0.49 | 0.6232 |
| **Gift card $200** | 2.84 | 0.0047 |
| **Required by employer** | 3.46 | 0.0006 |
| **Pay weekly COVID-19 test** | 0.83 | 0.4041 |
| **Increased insurance cost** | 3.68 | 0.0003 |
| **More transmissible and dangerous variant** | 7.32 | 0.000 |

*Notes:* For each incentive/disincentive scenario, we assigned the values “1” to those who are more likely to get vaccinated, “-1” to those who are less likely to get vaccinated, and “0” to those that report no change in intentions. We tested the hypothesis that the average change in intentions is significantly different from zero. Weighted t-tests.
