# Supplementary material for: Factors underlying COVID-19 vaccine and booster hesitancy and refusal, and incentivizing vaccine adoption
Source: PLoS One. 2022 Sep 22;17(9):e0274529. doi: 10.1371/journal.pone.0274529 (PMC9498968; doi:10.1371/journal.pone.0274529)
Supplement: S2 File — (DOCX) [file pone.0274529.s002.docx]

**WEB SURVEY OF COVID-19 VACCINE PREFERENCES IN THE UNITED STATES**

**CONSENT FORM**

**COVID-19 VACCINE PREFERENCES WEB SURVEY**

**Neil G. Bennett, David E. Bloom, Maddalena Ferranna**

**Version: August 25, 2021**

TERMS AND CONDITIONS:

You are asked to take part in a not-for-profit research study.

The purpose of this study is to gather information on COVID-19 vaccine preferences among the U.S. population. Specifically, we are interested in learning which factors affect people’s decisions to get or not to get vaccinated against COVID-19 and whether people display a preference for specific attributes of the vaccine. You are being asked to participate in this survey because you are 18 years of age or older and currently live in the United States.

Your participation in this study is voluntary and you may withdraw your participation at any time for any reason.

If you consent to participate, you will be asked a few questions about your age, gender, health, education, income, work, religion, political affiliation, views about COVID-19 vaccines and experience with the COVID-19 pandemic. The survey should take about 12 minutes to complete.

The possible risks of participating in this study include anxiety and stress caused by some of the questions. However, we foresee only minimal risks. There is also a risk of failure of confidentiality leading to your information being revealed. To minimize this risk, we will not ask for personal identifiers.

There are no direct benefits from participating in this study. You will be compensated the amount you agreed upon before entering the survey.

The information you provide will be completely confidential. We will only use the information to get a better picture of views on COVID-19 vaccines in the United States. You can decline to participate in any part of this study for any reason.

The data collected in this study will be held indefinitely, and may be used in future research projects related to the COVID-19 crisis.

If you are interested in seeing the overall results of the study, you will find them posted to <https://www.hsph.harvard.edu/pgda/data/> on or before November 1, 2021.

If you have any questions about this study, you can contact the research team at the following address: [Covid19.vaccine.preferences@gmail.com](mailto:Covid19.vaccine.preferences@gmail.com).

Thank you again for your time and participation.

**If you are experiencing stress or anxiety:**

The Centers for Disease Control (CDC) has resources and advice for managing stress and anxiety, including information about how to reduce the stress on yourself and others.

<https://www.cdc.gov/coronavirus/2019-ncov/daily-life-coping/managing-stress-anxiety.html>

The **Disaster Distress Helpline, 1-800-985-5990,** is a 24/7, 365-day-a-year, national hotline dedicated to providing immediate crisis counseling for people who are experiencing emotional distress related to any natural or human-caused disaster, including disease outbreaks like COVID-19. This toll-free, multilingual, and confidential crisis support service is available to all residents in the United States and its territories.

This research has been reviewed by a Harvard Longwood Medical Area Institutional Review Board (HMS/HSDM or HSPH) and by the City University of New York Institutional Review Board (CUNY IRB). If you wish to speak with someone from the IRB, please contact the Office of Human Research Administration (OHRA) at 617-432-2157 (or toll-free at 1-866-606-0573) or 90 Smith Street, Boston, Massachusetts 02120, or the CUNY Research Compliance Administrator at 646-664-8918 or email [HRPP@cuny.edu](mailto:HRPP@cuny.edu).

Do you consent to participate in this survey?

- Yes
- No

**SURVEY QUESTIONS**

We would now like you to answer a few background questions.

Q1. What is your gender?

- - Male
  - Female
  - Other

Q2. What is your age? _____

Q3. What is your race? (Select all that apply)

- - White
  - Black / African American
  - American Indian or Alaska Native
  - Asian
  - Native Hawaiian or Pacific Islander
  - Other

Q4. Are you of Hispanic or Latino origin?

- - Yes
  - No

Q5. What is the highest level of schooling that you have completed?

- - Less than high school degree
  - High school degree or GED
  - Some college or 2-year associate degree
  - 4-year college degree
  - Some postgraduate schooling, but no postgraduate degree
  - Master's degree
  - M.D., J.D., Ph.D., or some other doctoral degree

Q6. Where in the U.S. do you currently reside? (provide list)

Q7. Which comes closest to describing the area in which you live?

- - Urban
  - Suburban
  - Rural
  - I'm not sure

Q8. Whom do you live with? (Check all that apply.)

- - Partner (spouse, significant other, etc.)
  - Other family members
  - Friends or others unrelated to me
  - No one

DISPLAY Q9 ONLY IF Q8=”Partner” OR Q8=”Other family members” OR Q8=”Friends or other unrelated to me”.

Q9. Are any of your household members age 17 or under?

- - Yes
  - No
  - I’m not sure

DISPLAY Q10 ONLY IF Q8=”Partner” OR Q8=”Other family members” OR Q8=”Friends or other unrelated to me”.

Q10. Are any of your household members age 60 or above (not including you, if you’re 60 or above)?

- - Yes
  - No
  - I’m not sure

We now would like you to answer some questions about your health, your experience with COVID-19 and the experience of people close to you.

Q11. Have you ever tested positive for COVID-19?

- Yes
- No
- I’m not sure

DISPLAY QUESTION Q12 ONLY IF Q11=”Yes”

Q12. Have you ever been hospitalized due to the COVID-19 infection?

- Yes
- No

Q13. Do you have any of the following medical conditions? Check all that apply:

- - Cancer in the past 5 years (not including minor skin cancers)
  - Chronic kidney disease or dialysis
  - Chronic lung disease or moderate to severe asthma
  - High blood pressure
  - Stroke or transient ischemic attack
  - Heart disease (including heart attack or heart failure)
  - Diabetes (Type 1 or Type 2)
  - Obesity
  - Liver Disease
  - Immunocompromised state (i.e., take immune-suppressing drugs such as steroids, being treated for autoimmune disease, or had a solid organ transplant)
  - Pregnancy
  - Neurologic conditions
  - None that I am aware of

Q14. Prior to the availability of COVID-19 vaccines, what do you believe was your level of risk for getting infected with COVID-19?

- - High risk
  - Moderate risk
  - Low risk
  - No risk
  - I’m not sure

Q15. Has someone close to you (e.g., someone in your household, a family member outside your household, a friend, or a co-worker) been hospitalized with or died from COVID-19?

- - Yes
  - No
  - I’m not sure

Q16. Do you personally know anyone who has been vaccinated for COVID-19?

- Yes
- No
- I’m not sure

DISPLAY QUESTION Q17 ONLY IF Q16=”Yes”

Q17. Who was this person? (Check all that apply)

- Someone in your household
- A family member outside your household or a friend
- A co-worker
- Someone else (e.g., a neighbor, local shopkeeper, etc.)

DISPLAY QUESTION Q18 ONLY IF Q16=”Yes”

Q18. Would you say any of them had severe side effects from the vaccine?

- - Yes
  - No
  - I’m not sure

Q19. Do you typically get a flu vaccine each year?

- - Yes
  - No
  - I’m not sure

Q20. Are you currently covered by any form of health insurance?

- - Yes
  - No
  - I’m not sure

We now would like you to answer a few questions about your satisfaction with life.

Q21. Think back to January 2020, just before the COVID-19 pandemic began. On a scale from 0 to 10, rate your quality of life *at that time*, where 0 represents the worst possible life for you and 10 represents the best possible life for you:

**|-------|-------|-------|-------|-------|-------|-------|-------|-------|-------|**

**0 1 2 3 4 5 6 7 8 9 10**

**Worst Best**

**Possible Possible**

**Life Life**

Q22. How would you rate your *life right now*, where 0 represents the worst possible life for you and 10 represents the best possible life for you:

**|-------|-------|-------|-------|-------|-------|-------|-------|-------|-------|**

**0 1 2 3 4 5 6 7 8 9 10**

**Worst Best**

**Possible Possible**

**Life Life**

Q23. Thinking ahead *five years*, how would you expect to rate your life, where 0 represents the worst possible life for you and 10 represents the best possible life for you:

**|-------|-------|-------|-------|-------|-------|-------|-------|-------|-------|**

**0 1 2 3 4 5 6 7 8 9 10**

**Worst Best**

**Possible Possible**

**Life Life**

We now would like you to answer a few questions about your work.

Q24. What is your current employment status?

- - Employed full-time
  - Employed part-time
  - Not employed, but looking for a job
  - Not employed and not looking for a job

DISPLAY Q25 ONLY IF Q24=”Employed full-time” or Q24=”Employed part-time”.

Q25. Where are you currently working from?

- - Working from home
  - Working in a location outside my home
  - Combination of both

Q26. How has your total income changed during the pandemic?

- - It increased significantly
  - It decreased significantly
  - It did not change significantly
  - I’m not sure

Q27. Did you receive unemployment insurance benefits during the pandemic?

- Yes
- No
- I’m not sure

We would like you to answer some questions about mask wearing.

Q28. During the pandemic, but before COVID-19 vaccines were available to you, did you wear a mask when interacting with non-household members outside your home?

- - Always
  - Often
  - Sometimes
  - Rarely
  - Never
  - I’m not sure

DISPLAY Q29 ONLY IF Q28=”Always” OR Q28=”Often” OR Q28=”Sometimes” OR Q28=”Rarely”.

Q29. What has been your experience wearing a mask?

- Very uncomfortable
- Somewhat uncomfortable
- Not a problem

We would like you to answer some questions about your vaccination status now.

Q30. Have you been partially or fully vaccinated for COVID-19?

- - Yes
  - No

DISPLAY Q31 ONLY IF Q30=”Yes”

Q31. Which vaccine did you receive?

- - J & J
  - Both shots of Pfizer or Moderna
  - One shot of Pfizer or Moderna, and I am planning to get the 2^nd^ dose soon
  - One shot of Pfizer or Moderna, and I haven’t yet decided whether to get the 2^nd^ dose
  - One shot of Pfizer or Moderna, and I won’t get the 2^nd^ dose
  - Other

DISPLAY Q32 ONLY IF Q30=”Yes”

Q32. Did you have any side effects from the vaccine?

- I had severe side effects
- I had moderate side effects
- I had mild side effects
- I had no side effects
- I'm not sure

DISPLAY Q33 ONLY IF Q30=”Yes”

Q33. Do you regret having gotten the vaccine?

- - Yes. My side effects were severe and I wouldn't have taken the vaccine had I known I'd experience that reaction
  - Yes. Since I received my vaccination, I read/heard information that made me question the vaccine's long-term safety
- Yes. My confidence in the efficacy of the vaccines has decreased since the CDC has recommended booster shots.
  - Yes, I regret having gotten the vaccine for other reasons
  - No, now that the Pfizer COVID-19 vaccine has been *fully approved* by the FDA as opposed to being licensed by the FDA for *emergency use only*, I feel that it was a good decision to get vaccinated.
  - No, I have felt sure for a while now that it was a good decision to get vaccinated.
  - I'm not sure

DISPLAY Q34 ONLY IF Q31=” J & J” OR Q31=”Both shots of Pfizer or Moderna” OR Q31=”One shot of Pfizer or Moderna, and I am planning to get the 2^nd^ dose soon” OR Q31=”One shot of Pfizer or Moderna, and I haven’t yet decided whether to get the 2^nd^ dose” OR Q31=”Other”

Q34. Booster vaccines are being developed to address decreasing effectiveness over time of the original vaccine and the spread of new COVID-19 variants. For some vaccines, the Centers for Disease Control and Prevention now recommends a booster shot for COVID-19. Would you be willing to take the booster shot when it is available to you?

- Definitely yes
  - Probably yes
  - Probably no
  - Definitely no
  - I’m not sure

DISPLAY Q35 ONLY IF Q30=”No”

Q35. Do you intend to get vaccinated?

- - Definitely yes
  - Probably yes
  - Probably no
  - Definitely no
  - I’m not sure

Q36. The Pfizer COVID-19 vaccine has now been *fully approved* by the FDA as opposed to being licensed by the FDA for *emergency use only*. Does that increase your confidence in the effectiveness and safety of the vaccine?

- - Yes, a lot
  - Yes, a little
  - Probably no
  - Definitely no
  - I’m not sure

Now, we would like you to answer some questions about your reasons for being or not being vaccinated.

Q37. Which are the most important factors that make you think it is good to get vaccinated? Select all that apply. **(Qualtrics: Please randomize order of factors (other than “Other reasons” and “I don’t think there is any good reason to get vaccinated,” which should remain last).)**

- I’m afraid of hospitalization due to the virus
- I’m afraid of long-term illness (e.g., including “long-COVID”) due to the virus
- I’m afraid of death due to the virus
- I want my family to be protected from the virus
- I believe it’s our public duty to get vaccinated
- I’m concerned about traveling using subways, buses, airplanes, taxis, etc.
- My workplace requires or may require the vaccine
- I think I may be required to get the vaccine in order to get into restaurants or bars, sporting/entertainment events, etc.
- Other reasons
- I don’t think there is any good reason to get vaccinated

Q38. Which are the most important factors that make you think it is either unnecessary or a bad idea to get vaccinated? Select up to 8 factors. **(Qualtrics: Please randomize order of factors (other than “Other reasons,” and “There is no good reason to be unvaccinated,” which should remain last). Also, please alert respondent if s/he selects more than 8 factors and instruct her/him to limit choices to 8.)**

- I'm concerned about the side effects of the vaccine
- I'm concerned about the long-term safety of the vaccine
- I'm concerned that the vaccine was developed too quickly
- I don’t trust the government, the CDC, or the healthcare system
- I prefer to use masks and other precautions instead
- I don’t like vaccines in general
- I don't like needles
- I'm not at high risk for COVID-19
- I don't think COVID-19 is a serious illness
- I don't think the vaccine will work
- I believe the vaccine could give me COVID-19
- I had COVID-19 and I believe I’m immune
- I'm concerned about the costs associated with the vaccine (such as costs associated with an office visit or the vaccine itself)
- It's too inconvenient for me to get the vaccine (for example, due to the travel it would require or interference with my work hours)
- I believe my health and safety are in God's hands
- Other reasons
- There is no good reason to be unvaccinated

**INSTRUCTIONS TO QUALTRICS: FOR A GIVEN RESPONDENT, RANDOMLY SELECT ONE OF THE FOLLOWING REWARDS: $100 OR $200. THIS SINGLE RANDOM SELECTION APPLIES TO BOTH Q39 AND Q40.**

DISPLAY Q39 ONLY IF Q35=”Probably yes” OR Q35=”I’m not sure” OR Q35=”Probably no” OR Q35=”Definitely no” OR Q31=”One shot of Pfizer or Moderna, and I haven’t yet decided whether to get the 2^nd^ dose” OR Q31=”One shot of Pfizer or Moderna, and I won’t get the 2^nd^ dose”.

Q39. If you were offered a $100 ($200) gift card in exchange for being vaccinated for COVID-19, would you then be willing to be fully vaccinated?

- - Definitely yes
  - Probably yes
  - Probably no
  - Definitely no
  - I’m not sure

DISPLAY Q40 ONLY IF Q35=”Probably yes” OR Q35=”I’m not sure” OR Q35=”Probably no” OR Q35=”Definitely no” OR Q31=”One shot of Pfizer or Moderna, and I haven’t yet decided whether to get the 2^nd^ dose” OR Q31=”One shot of Pfizer or Moderna, and I won’t get the 2^nd^ dose”.

Q40. If, instead, you were offered $100 ($200) worth of tickets for a $1 million lottery in exchange for being vaccinated for COVID-19, would you then be willing to be fully vaccinated?

- - Definitely yes
  - Probably yes
  - Probably no
  - Definitely no
  - I’m not sure

DISPLAY Q41 ONLY IF Q35=”Probably yes” OR Q35=”I’m not sure” OR Q35=”Probably no” OR Q35=”Definitely no” OR Q31=”One shot of Pfizer or Moderna, and I haven’t yet decided whether to get the 2^nd^ dose” OR Q31=”One shot of Pfizer or Moderna, and I won’t get the 2^nd^ dose”.

Q41. If your employer required proof of COVID-19 vaccination as a (legal) condition of continued employment, would you then be willing to be fully vaccinated?

- - Definitely yes
  - Probably yes
  - Probably no
  - Definitely no
  - I’m not sure

DISPLAY Q42 ONLY IF Q35=”Probably yes” OR Q35=”I’m not sure” OR Q35=”Probably no” OR Q35=”Definitely no” OR Q31=”One shot of Pfizer or Moderna, and I haven’t yet decided whether to get the 2^nd^ dose” OR Q31=”One shot of Pfizer or Moderna, and I won’t get the 2^nd^ dose”.

Q42. Suppose your employer required all unvaccinated employees to be tested once a week for COVID-19 infection. You would be required to pay $10 for each weekly test. Would you then be willing to be fully vaccinated

- Definitely yes
  - Probably yes
  - Probably no
  - Definitely no
  - I’m not sure

DISPLAY Q43 ONLY IF Q35=”Probably yes” OR Q35=”I’m not sure” OR Q35=”Probably no” OR Q35=”Definitely no” OR Q31=”One shot of Pfizer or Moderna, and I haven’t yet decided whether to get the 2^nd^ dose” OR Q31=”One shot of Pfizer or Moderna, and I won’t get the 2^nd^ dose”.

Q43. Suppose the cost to you of health insurance increases by $200 per month if you are not fully vaccinated.  Would you become fully vaccinated to avoid that increase in monthly cost?

- - Definitely yes
  - Probably yes
  - Probably no
  - Definitely no
  - I’m not sure
  - I don’t have health insurance

DISPLAY Q44 ONLY IF Q35=”Probably yes” OR Q35=”I’m not sure” OR Q35=”Probably no” OR Q35=”Definitely no” OR Q31=”One shot of Pfizer or Moderna, and I haven’t yet decided whether to get the 2^nd^ dose” OR Q31=”One shot of Pfizer or Moderna, and I won’t get the 2^nd^ dose”.

Q44. If a substantially more transmissible and dangerous variant of COVID-19 began to circulate widely in the US, would you then be willing to be fully vaccinated?

- - Definitely yes
  - Probably yes
  - Probably no
  - Definitely no
  - I’m not sure

DISPLAY Q45 ONLY IF Q35=”Definitely yes” OR Q35=”Probably yes” OR Q35=”I’m not sure” OR Q35=”Probably no” OR Q31=” One shot of Pfizer or Moderna, and I am planning to get the 2^nd^ dose soon” OR Q31=”J&J” OR Q31=”Both shots of Pfizer or Moderna” OR Q31=”Other” OR Q31=”One shot of Pfizer or Moderna, and I haven’t yet decided whether to get the 2^nd^ dose”.

**INSTRUCTIONS TO QUALTRICS: FOR A GIVEN RESPONDENT, RANDOMLY SELECT ONE OF THE FOLLOWING OPTIONS: (55%, 75%), OR (55%, 95%), OR (75%, 95%). VACCINE A IS ASSIGNED THE LOWER PERCENTAGE EFFECTIVENESS AND VACCINE B IS ASSIGNED THE HIGHER PERCENTAGE EFFECTIVENESS. THIS ONE RANDOM SELECTION APPLIES TO Q45 THROUGH Q52.**

**Now we will describe two scenarios and ask you to compare different possible COVID-19 vaccines. In thinking about the scenarios, imagine yourself *in March 2020, at the beginning of the COVID-19 pandemic.***

**SCENARIO 1:**

**Suppose in this first scenario there are two COVID-19 vaccines, neither of which has side effects, and that no other COVID-19 vaccine will be available in the future.**

**Suppose further that Vaccine A is [55 or 75]% effective at reducing the risk of infection and is available immediately. On the other hand, Vaccine B is [75 or 95]% effective at reducing the risk of infection and will be available at a specific time in the future.**

**Finally, suppose both Vaccine A and Vaccine B require 2 shots.**

Q45. Would you be willing to wait at least 6 months for Vaccine B (the more effective vaccine) to become available and to take Vaccine B then? If you decide to wait for vaccine B, you don’t take vaccine A in the meantime.

- Yes
- No

DISPLAY Q46 ONLY IF Q45=”Yes”.

Q46. Choose, in the following range of 6 to 12 months, the longest you would be willing to wait for Vaccine B (the more effective vaccine) to become available.

**|----------|--------|---------|--------|---------|---------|**

**6 7 8 9 10 11 12**

**Maximum number of months willing to wait for Vaccine B**

DISPLAY Q47 ONLY IF Q45=”No”.

Q47. Would you immediately take Vaccine A (the less effective vaccine) (and not wait at all for Vaccine B, the more effective vaccine)?

- Yes
- No

DISPLAY Q48 ONLY IF Q47=”No”.

Q48. Choose, in the following range of 1 to 5 months, the longest you would be willing to wait for Vaccine B (the more effective vaccine) to become available.

**|----------|--------|---------|--------|**

**1 2 3 4 5**

**Maximum number of months willing to wait for Vaccine B**

DISPLAY Q49 ONLY IF Q35=”Definitely yes” OR Q35=”Probably yes” OR Q35=”I’m not sure” OR Q35=”Probably no” OR Q31=” One shot of Pfizer or Moderna, and I am planning to get the 2^nd^ dose soon” OR Q31=”J&J” OR Q31=”Both shots of Pfizer or Moderna” OR Q31=”Other” OR Q31=”One shot of Pfizer or Moderna, and I haven’t yet decided whether to get the 2^nd^ dose”.

**SCENARIO 2:**

**Suppose in this second scenario that, once again, there are two COVID-19 vaccines, neither of which has side effects, and that no other COVID-19 vaccine will be available in the future.**

**Suppose further that Vaccine A is [55 or 75]% effective at reducing the risk of infection and is available immediately. On the other hand, Vaccine B is [75 or 95]% effective at reducing the risk of infection and will be available at a specific time in the future.**

**This time, however, suppose Vaccine A requires only 1 shot and will be fully effective after 2 weeks. Vaccine B requires 2 shots and will be fully effective 6 weeks after the first dose.**

Q49. Would you be willing to wait at least 6 months for Vaccine B (the more effective, 2-shot vaccine) to become available and to take Vaccine B then? If you decide to wait for vaccine B, you don’t take vaccine A in the meantime.

- Yes
- No

DISPLAY Q50 ONLY IF Q49=”Yes”

Q50. Choose, in the following range of 6 to 12 months, the longest you would be willing to wait for Vaccine B (the more effective, 2-shot vaccine) to become available.

**|----------|--------|---------|--------|---------|---------|**

**6 7 8 9 10 11 12**

**Maximum number of months willing to wait for Vaccine B**

DISPLAY Q51 ONLY IF Q49=”No”

Q51. Would you immediately take Vaccine A (the less effective, 1-shot vaccine) (and not wait at all for Vaccine B, the more effective, 2-shot vaccine)?

- Yes
- No

DISPLAY Q52 ONLY IF Q51=”No”

Q52. Choose, in the following range of 1 to 5 months, the longest you would be willing to wait for Vaccine B (the more effective, 2-shot vaccine) to become available.

**|----------|--------|---------|--------|**

**1 2 3 4 5**

**Maximum number of months willing to wait for Vaccine B**

DISPLAY Q53 ONLY IF Q35=”Definitely yes” OR Q35=”Probably yes” OR Q35=”I’m not sure” OR Q35=”Probably no” OR Q31=” One shot of Pfizer or Moderna, and I am planning to get the 2^nd^ dose soon” OR Q31=”J&J” OR Q31=”Both shots of Pfizer or Moderna” OR Q31=”Other” OR Q31=”One shot of Pfizer or Moderna, and I haven’t yet decided whether to get the 2^nd^ dose”.

**INSTRUCTIONS TO QUALTRICS: PLEASE RANDOMIZE THE ORDER OF THE FOUR OPTIONS IN Q53**

Q53. From 1 to 4, how would you rank the importance of the following four characteristics of COVID-19 vaccines, where 1 is the most important characteristic and 4 the least important one?

- Effectiveness of the vaccine at protecting from infection
- How quickly the vaccine is available to you
- Mild side effects of the vaccine
- The vaccine requires only one shot instead of two

DISPLAY Q54 ONLY IF Q35=”Definitely yes” OR Q35=”Probably yes” OR Q35=”I’m not sure” OR Q35=”Probably no” OR Q31=” One shot of Pfizer or Moderna, and I am planning to get the 2^nd^ dose soon” OR Q31=”J&J” OR Q31=”Both shots of Pfizer or Moderna” OR Q31=”Other” OR Q31=”One shot of Pfizer or Moderna, and I haven’t yet decided whether to get the 2^nd^ dose”.

Q54. Suppose you now have a choice between 2 COVID-19 vaccines, Vaccine A and Vaccine B, both of which require 1 shot:

1. Vaccine A reduces the risk of infection and transmission, but if you do get infected, you may get severely ill.
2. Vaccine B does not reduce the risk of infection or transmission, but if you do get infected, you have only mild symptoms.

Which vaccine do you prefer?

- I strongly prefer Vaccine A
- I mildly prefer Vaccine A
- I don’t prefer one vaccine over the other
- I mildly prefer Vaccine B
- I strongly prefer Vaccine B

We are almost at the end of the survey. We would like you to answer a few more background questions now.

Q55. Have you consumed alcohol in the past two years?

- Yes
- No
- I’d rather not say

DISPLAY Q56 ONLY IF Q55=”Yes”.

Q56. How has your drinking of alcoholic beverages (e.g., beer, wine, cocktails) changed since the beginning of the COVID-19 pandemic?

- Increased a lot
- Increased moderately
- Unchanged
- Decreased moderately
- Decreased a lot
- I’m not sure

Q57. In 2020 (last year), what was your total household income (from all sources, before taxes)?

- - Less than $15,000
  - $15,000 to $24,999
  - $25,000 to $34,999
  - $35,000 to $49,999
  - $50,000 to $74,999
  - $75,000 to $99,999
  - $100,000 to $149,999
  - $150,000 to $199,999
  - $200,000 or more
  - I’m not sure
  - I’d rather not say

Q58. How would you *best* describe your religious affiliation?

- - Evangelical Christian
  - Non-Evangelical Christian
  - Jewish
  - Muslim
  - Other religion
  - None
  - I’m not sure
  - I’d rather not say

Q59. Do you consider yourself a Republican, Democrat, Independent, or other? (**INSTRUCTIONS TO QUALTRICS: PLEASE rotate R & D**)

- Republican
- Democrat
- Independent
- Other
- I’m not sure
- I’d rather not say

Q60. Whom did you vote for in the 2020 Presidential election? (**INSTRUCTIONS TO QUALTRICS: PLEASE rotate Biden & Trump**)

- Joseph R. Biden
- Donald J. Trump
- Someone else
- I did not vote
- I’d rather not say

Q61. What is your sexual orientation?

- Heterosexual or straight
- Gay
- Lesbian
- Bisexual
- Not listed above (please specify): _________
- I’d rather not say

Thank you for your responses. They are very helpful.
